# Intelligent Fault Diagnosis of Rolling Element Bearings Based on Modified AlexNet †

**DOI:** 10.3390/s23187764

**Published:** 2023-09-08

**Authors:** Mohammad Mohiuddin, Md. Saiful Islam, Shirajul Islam, Md. Sipon Miah, Ming-Bo Niu

**Affiliations:** 1 Department of Electronics and Telecommunication Engineering, Chittagong University of Engineering and Technology (CUET), Chittagong 4349, Bangladesh; muhin.cuet.bd@gmail.com (M.M.); saiful05eee@cuet.ac.bd (M.S.I.); shiraj.cuet@gmail.com (S.I.); 2 Department of Information and Communication Technology, Islamic University, Kushtia 7003, Bangladesh; sipon@iu.ac.bd; 3 Department of Signal Theory and Communications, University Carlos III of Madrid (UC3M), 28911 Madrid, Spain; 4 IVR Low-Carbon Research Institute, School of Energy and Electrical Engineering, Chang’an University, Xi’an 710064, China

**Keywords:** CNN model, discrete wavelet transform, global average pooling, intelligent fault diagnosis, vibration image

## Abstract

The reliable and safe operation of industrial systems needs to detect and diagnose bearing faults as early as possible. Intelligent fault diagnostic systems that use deep learning convolutional neural network (CNN) techniques have achieved a great deal of success in recent years. In a traditional CNN, the fully connected layer is located in the final three layers, and such a layer consists of multiple layers that are all connected. However, the fully connected layer of the CNN has the disadvantage of too many training parameters, which makes the model training and testing time longer and incurs overfitting. Additionally, because the working load is constantly changing and noise from the place of operation is unavoidable, the efficiency of intelligent fault diagnosis techniques suffers great reductions. In this research, we propose a novel technique that can effectively solve the problem of traditional CNN and accurately identify the bearing fault. Firstly, the best pre-trained CNN model is identified by considering the classification’s success rate for bearing fault diagnosis. Secondly, the selected CNN model is modified to effectively reduce the parameter quantities, overfitting, and calculating time of this model. Finally, the best classifier is identified to make a hybrid model concept to achieve the best performance. It is found that the proposed technique performs well under different load conditions, even in noisy environments, with variable signal-to-noise ratio (SNR) values. Our experimental results confirm that this proposed method is highly reliable and efficient in detecting and classifying bearing faults.

## 1. Introduction

The most crucial part of rotating machines is the bearing, whose main purposes are to sustain the mechanical rotating body and decrease the friction coefficient while it is in motion [1]. Rolling bearings are employed to transmit loads from moving to stationary components or vice versa as well as to create conditions for the relative movement of rotating parts [2,3]. Efficient bearing fault diagnosis is required to ensure the smooth operation of mechanical system. There are two parts to bearing fault identification problem. The initial part focuses on the extraction of fault information-related features from vibration signals, and the latter one on fault identification, which makes use of the extracted features for problem detection by applying a variety of artificial intelligence (AI) approaches, including an artificial neural network (ANN), a decision tree (DT), the k-nearest neighbors (k-NN) algorithm, a support vector machine (SVM), neuro-fuzzy [4,5,6,7,8,9,10,11], etc.

Samanta et al. [12] conducted a study comparing the effectiveness of three different ANN types for detecting bearing faults: multi-layer perception (MLP), the radial basis function (RBF) network, and the probabilistic neural network (PNN). The preprocessing of the data, manual feature extraction, and pattern detection are often necessary for the ANN classification approach. Features are extracted from those ANNs using the crest-factor, envelope spectrum, root mean square (RMS), crest value, standard deviation, Kurtosis, variance, and estimation [13]. Due to complex vibration signals, it is difficult for the manual feature extraction technique of ANN [14] to extract these features. Because a signal is impacted by surrounding noise as a result of changes in working situations, existing intelligent fault identification systems in real industries have limitations with initial features and multiple unidentified complex failure causes. The ANN model does not produce sufficient results for the reason that the method of extracting features depends on a high level of professional skill.

To overcome the limitations of the existing intelligent approach, the deep learning (DL) method has recently been used and produced satisfactory results. DL approaches have the benefit of automatically learning characteristic features and challenging nonlinear relationships from raw data [15,16]. However, its application in bearing fault detection is still being developed [17]. One of the deep learning models is the deep convolutional neural network (DCNN), which is an effective tool for two-dimensional image processing [18]. It is resilient, can be trained on large amounts of data, and is unaffected by image distortion [19]. A new DL model based on a DCNN for detecting bearing problems in induction motors (IMs) was proposed by Khan Ma et al. [20]. In addition, DCNN provides advantages such as quick inference, and the capacity to encode richer, and higher-order network topologies. Because of its high classification accuracy, DCNN has found widespread use in computer vision [21]. SVM and CNN are combined by Shao, Y. et al. [22] to present a novel hybrid intelligent fault diagnostic frame that is superior to some conventional fault diagnosis techniques and exhibits high precision for rolling bearings. Time domain signals are converted to two-dimensional (2D) images in several studies of fault diagnosis, utilizing 2D forms of signals [23,24]. Although 2D image-based bearing fault diagnosis has achieved excellent accuracy, its performance is still mostly dependent on handmade feature extraction.

Currently, the pre-trained model is the most widely used convolutional neural network and has become one of the hotspots in bearing fault diagnosis. Bearing fault signals are complex due to the high variance, nonlinear, and nonstationary characteristics of vibration signals [12]. As a result, the input distributions of the layers of the pre-trained model differ from each other. This can make achieving high accuracy in parameter training very challenging and time-consuming, which requires proper setup [25]. In a traditional AlexNet, the fully connected (FC) layer is located in the final three layers. An FC layer consists of multiple layers that are all connected [26]. It can be defined as a function from *Rm* to *Rn*. Each input parameter of each layer influences each output parameter. However, the FC layer of the AlexNet has too many training parameters, which makes the model training and testing time longer, and causes overfitting.

To address the aforementioned problems, this work develops a novel technique for the intelligent identification of bearing faults in rotating machinery. It effectively reduces training parameters, solves the traditional AlexNet model’s overfitting problem, and also increases the classification accuracy. In the proposed technique the best pre-trained CNN model is firstly selected for the bearing fault diagnosis. In the next step, the selected CNN model is improved by replacing the FC layer with a global average pooling (GAP) layer adding some batch normalization (BN) layers to prevent this internal covariate shifting, which effectively decreases the parameter quantity, overfitting, and calculating time of the CNN model. The last step constructs a hybrid model concept to achieve the best performance.

## 2. Proposed Methods and Materials

**Figure 1:**
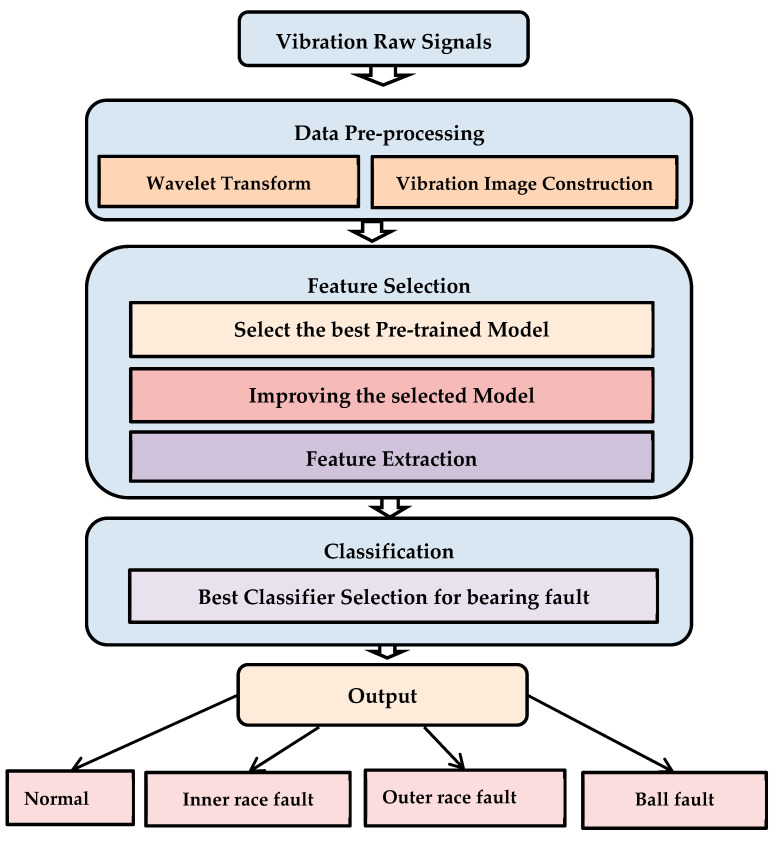
Flowchart of the proposed system.

Due to DCNN’s disadvantage of having too many training parameters in an FC layer, model testing and training take more time so that overfitting occurs. Additionally, the performance of intelligent defect diagnosis procedures is severely hampered by the continually changing operating load and inevitable noise from the environment. To solve the difficulty of the existing intelligent method, this study proposes a novel technique. This method involves an effective data pre-processing technique, feature selection technique, and best classifier selection technique to detect and classify the bearing signals. In the proposed method we firstly classify the bearing fault by considering the original dataset through GoogleNet, AlexNet, ResNet50, and VGG-16. In the next step, because of the classification’s success rate of AlexNet compared to other pre-trained models, we modify the traditional AlexNet model structure, which effectively reduces the parameter quantity, overfitting, and calculation time of the CNN model. In the last step of the experiment, the deep features obtained from the last layer of the proposed modified model are applied separately as the input Softmax, k-NN, and SVM classifier and select the best classifier, which effectively improves the accuracy of the classification results. The flowchart of the proposed method is illustrated in Figure 1.

### 2.1. Data Pre-Processing

To develop an effective data pre-processing technique, one uses wavelet transform and vibration image construction processes.

#### 2.1.1. Wavelet Transform

**Figure 2:**
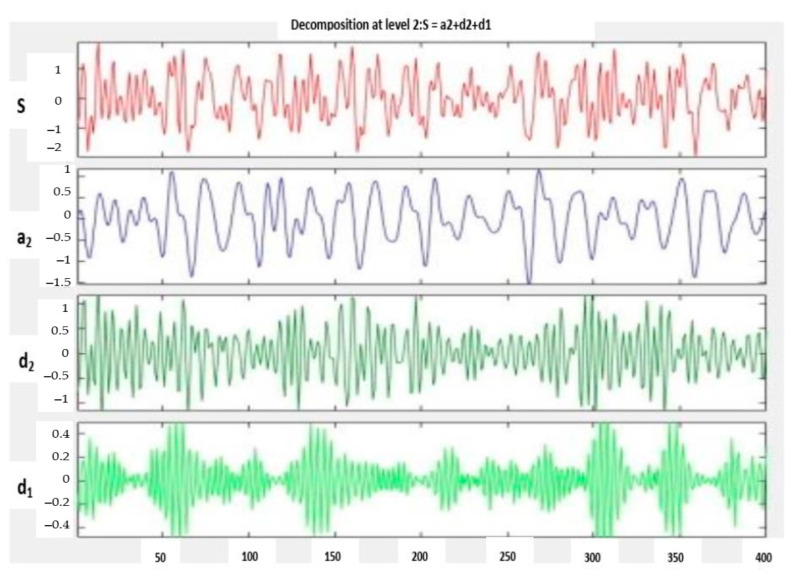
Output result of wavelet transforms.

The real working environments have noise that contaminates the sensory inputs. The noise removal from the data is required before proceeding with further data analysis. Traditional Fourier transform (FT) is appropriate for stationary signals. However, information can often be observed in the frequency domain that is not easily seen in the time domain. It is critical to obtain the time-frequency characteristics of non-stationary signals. Wavelet transform (WT) is an excellent choice for processing such signals [27]. A complicated unsteady signal in terms of frequency or time-domain can be represented by a wavelet transform. The discrete wavelet transform (DWT) is a signal decomposition technique that employs a collection of distinct, spatially aligned frequency bands. Dual filters handle the vibration signal, producing two signals: details and approximation. This technique is known as signal analysis or signal decomposition. The apparatuses of the breakdown signal can be further rebuilt into the original raw signal without losing any information [28]. In this study, one-dimensional wavelet decomposition was used up to two levels to represent the complexity of unsteady vibration signals. The outcome of wavelet decomposition is shown in Figure 2, which depicts the selection of two levels of detail (d1, d2) and approximation (a2) for each signal.

#### 2.1.2. Vibration Image Construction

**Figure 3:**
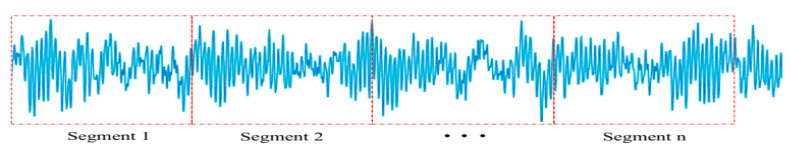
Signal data segmentation.

**Figure 4:**
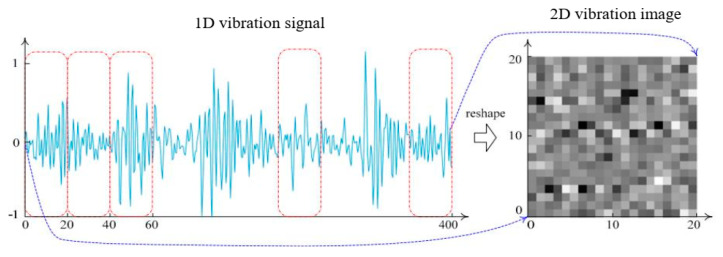
Vibration image construction.

**Figure 5:**
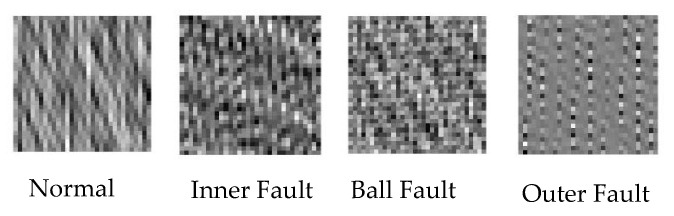
Vibration image type.

CNN is initially ideal for processing 2D inputs because of three important architectural ideas: local receptive fields, weight sharing, and spatial polling [29]. It is significantly easier to extract information from high-dimensional data, yet bearing vibration signals are 1D data. Motivated by this fact, time-domain vibration signals were transformed into 2D gray images. The decomposed vibration signals were separated into segments of the same length. The procedure for segmenting signals is shown in Figure 3. An image of vibration with a size of 20 × 20 was generated from one segment of a signal. The quantity of samples in the vibration signal was the same as the number of pixels in the vibration image. The process for generating a vibration image is shown in Figure 4. Accordingly, we obtained four different vibration image types, as shown in Figure 5.

### 2.2. Feature Selection

After constructing the vibration images, features were selected by the proposed modification of the AlexNet network. At first, pre-trained models, AlexNet, GoogleNet, VGG16, and ResNet50 were used to observe the bearing fault classification performance and AlexNet provided the highest accuracy. Then, AlexNet was modified by adding and replacing layers to obtain better accuracy. Finally, features were extracted from the modified AlexNet model.

#### 2.2.1. Best Pre-Trained Model Selection

**Figure 6:**
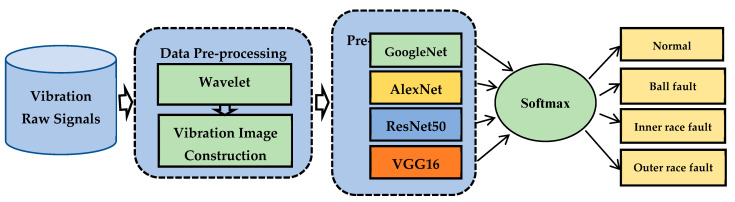
Best pre-trained model selection process.

In this subsection, the bearing fault was classified based on the vibration image using GoogleNet, AlexNet, ResNet50, and VGG-16. The procedure for selecting the best pre-trained model is shown in Figure 6. To train the pre-trained model, such as GoogleNet AlexNet, ResNet50, and VGG16, we introduced the last three layers, which were the FC layer, Softmax layer, and output layer. The ADAM and stochastic gradient descent (SGD) optimization techniques were examined during the training of the deep models. Table 1 shows the classification result of pre-trained models. AlexNet was selected for analyzing the model because it achieved the highest accuracy.

#### 2.2.2. Modified AlexNet Model

**Figure 7:**
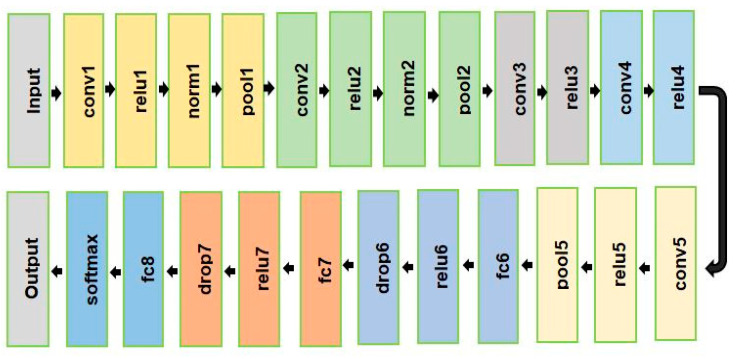
AlexNet model.

At present, AlexNet is the most widely used convolutional neural network and has become one of the hotspots in bearing fault diagnosis [17]. Due to some limitations of AlexNet, it is very challenging to achieve satisfactory results in bearing fault diagnosis. Figure 7 shows the traditional AlexNet model and the limitations of AlexNet are described below:

Bearing fault signals are complex due to the high variance, nonlinear, and nonstationary characteristics of vibration signals. As a result, the input distributions of the layers of AlexNet differ from each other and internal covariate shifting occurs [17]. This can make achieving accuracy in parameter training very challenging and time-consuming, which requires proper setup [30]. In a traditional AlexNet, the FC layer is located in the final three layers, namely fc6, fc7, and fc8. An FC consists of many layers that are all connected [31]. The FC layer of AlexNet has the disadvantage of too many training parameters. The procedure for calculating the training parameters of FC layers is given below. In AlexNet, there are two types of FC layers. The first FC layer (fc6) is connected to the final conv layer, whereas subsequent FC layers (fc7 and fc8) are connected to additional FC layers. Each situation is analyzed separately.

Case 1: An FC (fc6) layer’s number of parameters connected to a conv layer can be calculated [30] by the following equations

(1) $$P_{cf}=W_{cf}+B_{cf}$$

(2) $$B_{cf}=F$$

(3) $$W_{cf}=F\times N\times O^{2}$$

where:

*P_cf_* = number of parameters;
*W_cf_* = The number of weights in an FC layer that is linked to a conv layer;
*B_cf_* = The number of biases in an FC layer that is linked to a conv layer;
*O* = The size of the previous conv layer’s output image;
*N* = The number of kernels in the last conv layer;
*F* = The number of neurons in the FC layer.

In the first FC layer (fc6) of AlexNet, *F* is 4096, *N* is 256, and *O* is 6. Therefore,

$$\begin{array}{ll} W_{cf} & =4096\times 256\times 6^{2}\\ & =37,748,736 \end{array}$$

$$B_{cf}=4096$$

$$\begin{array}{ll} P_{cf} & =W_{cf}+B_{cf}\\ & =37,748,736+4096\;\\ & =37,\;752,832 \end{array}$$

Case 2: An FC layer’s number of parameters connected to an FC layer can be calculated [30] by the following equations

(4) $$P_{ff}=B_{ff}+W_{ff}$$

(5) $$B_{ff}=F$$

(6) $$W_{ff}=F_{-1}\times F$$

where:

$P_{cf}=$ number of parameters;
$W_{cf}=$ The number of weights in an FC layer that is linked to an FC layer;
$B_{ff}=$ The number of biases in an FC layer that is linked to an FC layer;
F = The number of neurons in the FC layer;
$F_{-1}=$ The number of neurons in the just before FC layer.

In the second FC layer (fc7) of AlexNet, *F* is 4096, and *F*_−1_ = 4096. Therefore,

$$B_{ff}=F\;=4096$$

$$W_{ff1}=F_{-1}\times F=4096\times 4096=16,777,216$$

$$\begin{array}{ll} P_{ff1} & =B_{ff}+W_{ff}=4096+16,777,216\\ & =16,781,312 \end{array}$$

In the last FC layer (fc8) of AlexNet, *F* is 1000, and *F*_−1_ = 4096. Therefore,

$$B_{ff}=F\;=1000$$

$$W_{ff}=F_{-1}\times F=4096\times 1000=4,096,000$$

$$\begin{array}{ll} P_{ff2} & =B_{ff}+W_{ff}=1000+4,096,000\\ & =4,097,000 \end{array}$$

The sum of the parameters in AlexNet’s three FC layers makes up the total amount of parameters

$$\begin{array}{ll} P_{total} & =P_{cf}+P_{ff1}+P_{ff2}\\ & =37,\;752,832+16,781,312+4,097,000\\ & =58,631,144 \end{array}$$

**Figure 8:**
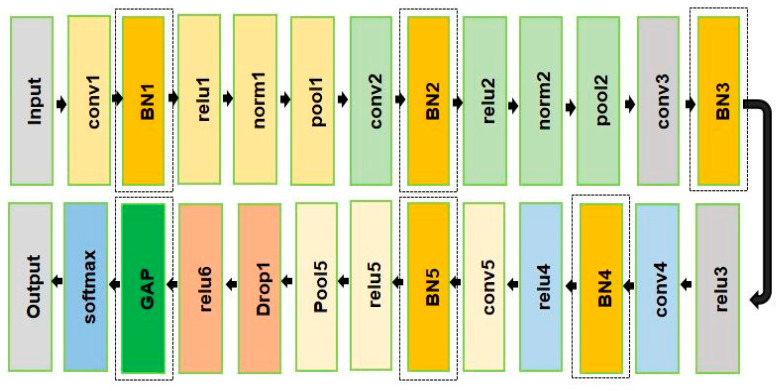
Modified AlexNet model.

After the calculation, it can be seen in Table 2 that there are 62,378,344 parameters in AlexNet but of them 58,631,144 training parameters came from the last three FC layers of AlexNet, which is a significant proportion. However, the FC layer of AlexNet has the disadvantage of too many training parameters, which makes the model training and testing time longer and incurs overfitting. This research modified the structure of the AlexNet model by analyzing the limitations of the traditional AlexNet model. Figure 8 shows the modified AlexNet model.

Firstly, the fully connected layer of AlexNet is replaced by the GAP, which effectively reduces the total number of parameters, training, and testing time, and also avoids overfitting. Secondly, the BN layer is adopted in the traditional AlexNet to prevent this internal covariate shifting. The concept of BN is straightforward. When CNNs are trained in mini batch mode, the normalization transform is applied to the layer activations by BN to maintain constant means and variances. It effectively makes good parameter training and *accelerates the* training time and accuracy.

Selecting the optimal AlexNet model hyper-parameters throughout the CNN model construction process can significantly increase the modified AlexNet model’s fault diagnostic accuracy, test speed, and training speed. The optimizer, activation functions, learning rates, convolution kernels, and pooling kernels are the primary hyper-parameters that have an important effect on the CNN model’s performance [20]. Table 3 shows the hyper-parameters of the benchmark model. The ADAM adaptive optimization technique is used in this model, and the learning rate can be modified adaptively.

The number of parameters in a convolutional layer in the modified AlexNet is given [30] by the following equations

(7) $$P_{c}=B_{c}+W_{c}$$

(8) $$B_{c}=N$$

(9) $$W_{c}=C\times N\times K^{2}$$

where *W_c_* = No. of weights, *B_c_* = No. of biases, *P_c_* = No. of parameters, *K* = width of kernels, *N* = No. of kernels, and *C* = No. of channels.

In the modified AlexNet, at the conv1 layer, *C* = 3, *K* = 11, *N* = 96.

$$\mathrm{So},\;B_{c}=N=96$$

$$\begin{array}{ll} W_{c} & =C\times N\times K^{2}\\ & =3\times 96\times 11^{2}\\ & =34848\; \end{array}$$

$$\begin{array}{ll} P_{c} & =B_{c}+W_{c}\\ & =96+34,848=34,944 \end{array}$$

Similarly, the number of parameters can be calculated for conv2, conv3, conv4, and conv5, and they are 614,656, 885,120, 1,327,488, and 884,992, respectively.

The total number of parameters of the modified AlexNet is summarized in Table 4. It can be seen that only 3,752,704 parameters are used in the modified AlexNet whereas 62,378,344 parameters were used in the traditional AlexNet. More than 58,625,640 (62,378,344–3,752,704) parameters were used in the traditional AlexNet, which makes the model training, and testing time longer, and occurs overfitting. In contrast, the proposed modified AlexNet effectively reduces the total number of parameters, training, and testing time, and it also avoids overfitting.

#### 2.2.3. Best Classifier Selection for Bearing Faults

This section presents the hybrid model concept. Additionally, to achieve the best classification performance, the features obtained from the last layer of the proposed modified AlexNet are applied as the input to various machine learning models, including Softmax, kNN, SVM, and DT. Furthermore, the classification abilities of the models are investigated individually.

## 3. Experimental Verification Based on Vibration Signals

### 3.1. Testbed Description

In this research, the proposed system has been employed to diagnose the experimental vibration signals of bearings to evaluate as well as verify the effectiveness and accuracy of intelligent diagnosis. Two datasets are used for evaluating the proposed system: the CWRU dataset, and the MFPT dataset [31,32].

**Figure 9:**
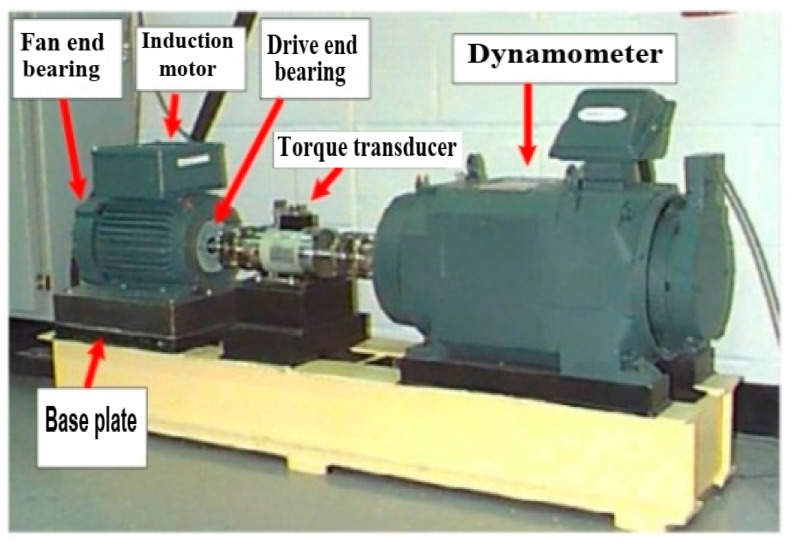
Experimental setup [31].

**Figure 10:**
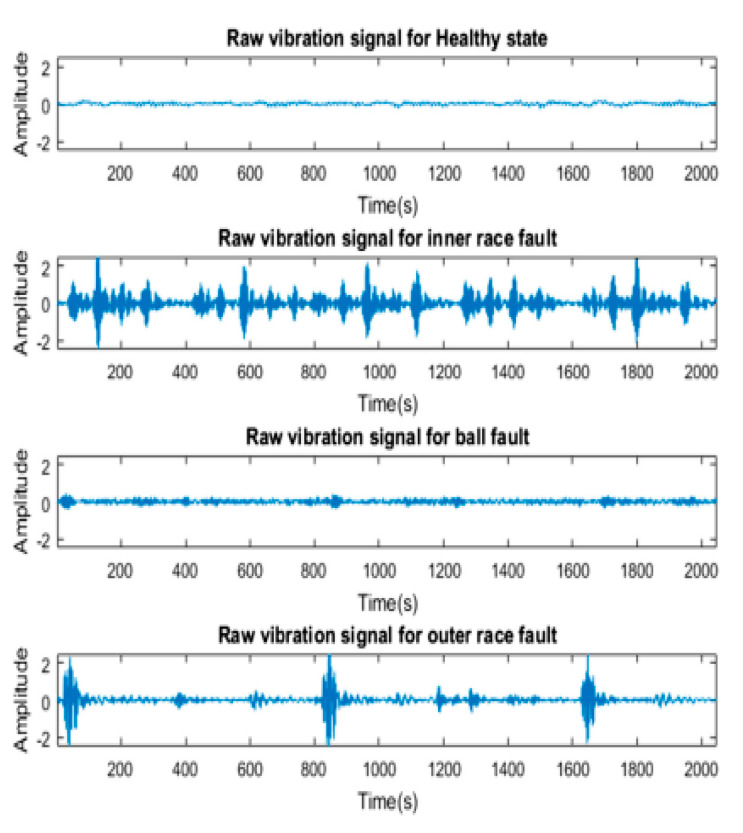
Raw vibration signals.

**The CWRU dataset:** This dataset was chosen for this analysis because CWRU data have received favorable reviews from many scholars studying bearing failure [4]. The four classes in this dataset are healthy (normal), inner race fault, ball fault, and outer race fault. Data on vibration signals are collected using accelerometers. The experimental setup, which includes a 1.5 KW (2 hp) induction motor, is shown in Figure 9. To obtain data, sampling frequencies of 12 kHz and 48 kHz were used. Deep groove ball bearings of the 6205-2RS JEM SKM type are considered to be the operational condition. In this work, 409,600 samples of data were taken into account for normal bearings and 409,600 samples are taken into account for fault data. The fault diameter in this study is 1.016 mm (0.014 inches). Figure 10 shows each of the four raw vibration signals.

**The MFPT dataset:** The society for machinery failure prevention technology (MFPT) dataset [32] is also used for rolling bearing fault evaluation and study. The MFPT bearing data make use of a NICE bearing. This dataset contains three types of bearing data: normal bearing data, inner race fault data, and outer race fault data at varying loads. The data are from a single-channel radial accelerometer. In this work, 409,600 samples of data are taken into account for normal bearings and 409,600 samples are taken into account for fault data.

### 3.2. Experimental Outcome

For testing reasons, a laptop with a Core i3-5005U processor was used. For the coding environment, the MATLAB 2020a version was utilized. Following the simulation, the results of training, validation, and testing were recorded.

#### 3.2.1. The Proposed System’s Performance

The proposed system was trained on 1024 vibration images and tested on 400 vibration images. Five BN layers were added, and the FC layer was replaced with the GAP layer to AlexNet. Table 5 shows the comparison between the FC layer, and GAP of five trials. It can be observed by comparing Table 5, that as a result of using FC, due to overfitting, the testing accuracy decreased from the training accuracy. In contrast, by using GAP instead of FC, overfitting was reduced. However, the accuracy was slightly reduced. Five BN layers were added to improve accuracy. The final proposed system’s training and testing accuracy is shown in Table 6. It can be observed by comparing Table 6 that the performance of the improved AlexNet model was much better than the performance of the traditional FC AlexNet model. The full connection layer is removed in the modified AlexNet model, which has a significant impact on the model’s ability to be used for online, quick fault diagnosis. This also had a big effect on the number of model parameters there are and the duration of the training required. The modified AlexNet model achieved an accuracy of 98.30%, whereas the traditional AlexNet model was 94.40% accurate [32]. The proposed modified AlexNet model’s overfitting problems are not present in our proposed method.

**Figure 11:**
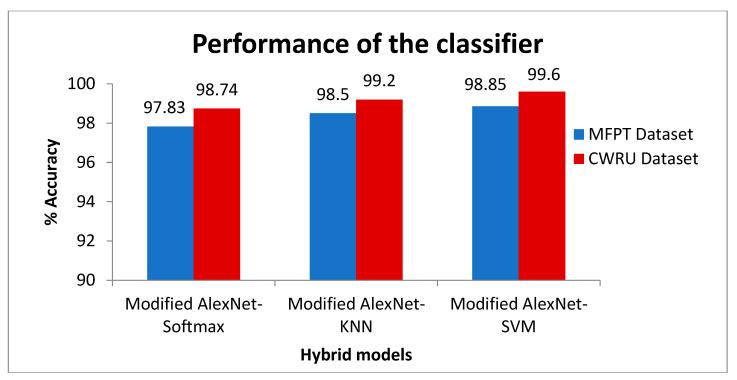
Performance of hybrid models.

This section presents the hybrid model concept. Additionally, to achieve the best classification performance, the features obtained from the last layer of the modified AlexNet are applied as the input to various machine learning models, including Softmax, kNN, and SVM. Furthermore, the classification abilities of the models are investigated individually. Figure 11 shows the performance of the classifier on the CWRU dataset and the MFPT dataset. The accuracy of the CWRU dataset is shown in red, whereas the accuracy of the MFPT dataset is represented in blue. It can be observed by comparing Table 5 that the performance of the modified AlexNet–SVM hybrid model is much better than the performance of the modified AlexNet–Softmax, and modified AlexNet–kNN hybrid models on both the CWRU dataset, and the MFPT dataset.

**Figure 12:**
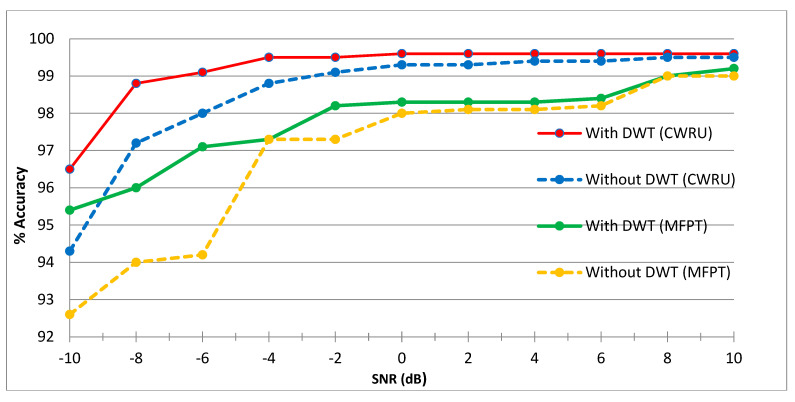
Impact on DWT.

This section compares the results of using DWT vs. without DWT in a noisy environment. Figure 12 shows how DWT impacted classification results. In Figure 12 the red line on the graph represents performance with DWT, while the blue dot line represents performance without DWT on the CWRU dataset and the green color line on the graph represents performance with DWT, while the yellow dot line represents performance without DWT on MFPT dataset. The noisy situation on the CWRU dataset is shown, and it is clear from the graph that using DWT is more effective than not using DWT in a noisy environment. On the MFPT dataset, the performance under noisy conditions with DWT is shown by the green color line, and the performance without DWT is shown by the yellow dot line. The graph shows that the proposed DWT model for a noisy environment performs better on the CWRU, and the MFPT datasets, respectively.

#### 3.2.2. Evaluation Measurements of the Proposed System

**Figure 13:**
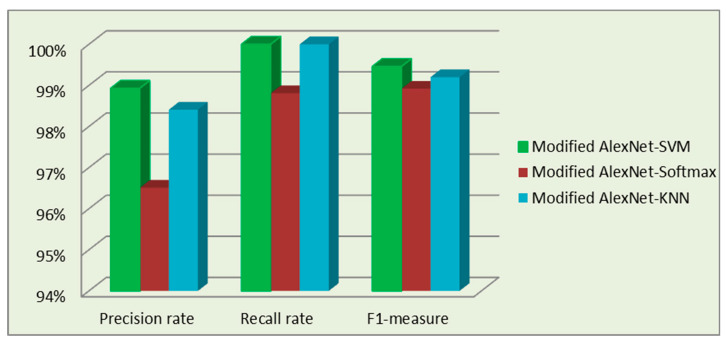
Evaluation results.

In this study, the precision ratio, recall ratio, and F1 measure are calculated to further investigate and analyze the proposed method’s performance in terms of classification. The probability of truly positive values out of all projected positive values is referred to as precision. Recall measures how often truly positive values are expected to be positive values. F1 represents the harmonic mean of recall and precision [31] as follows:

(10) $$\mathrm{Precision}\;\left(P\right)=\frac{\mathrm{TP}}{\left(\mathrm{TP}+\mathrm{FP}\right)}\;\;\;\;\;\;\;\;\;\;\;\;\;\;\;\;\;\;\;$$

(11) $$\mathrm{Recall}\;\left(R\right)=\frac{\mathrm{TP}}{\left(\mathrm{TP}+\mathrm{FN}\right)}\;\;\;\;\;\;\;\;\;\;\;\;\;\;\;\;\;\;\;\;$$

(12) $$F1=\frac{2\times \;\mathrm{TP}}{2\times \;\mathrm{TP}+\mathrm{FP}+\mathrm{FN}}\;\;\;\;\;\;\;\;\;\;\;\;\;\;\;\;\;\;\;$$

where TP is the number of actual positive events, FP is the number of false positives, and FN is the number of false negatives. Based on Equations (10)–(12), precision, recall, and F1 can be calculated on the experimental findings of the proposed system in Table 7. As shown in Figure 13, the precision rate is 98.93%, the recall rate is 100%, and the F1 measure is 99.46%.

#### 3.2.3. Evaluation in a Noisy Situation

Noise has an impact on signals in real applications in industry. Noise is an additional problem that is created because of changes in the working environment, which decrease the model’s performance. In the following section, the effectiveness of the proposed system is investigated in a noisy situation while identifying bearing faults. Before being tested on noisy signals, the proposed model is trained on the original signal. Additive white Gaussian (AWG) noise is added to create a noisy signal by changing the signal–noise ratio (SNR) to raw signals. The SNR is determined as follows:

(13) $$\mathrm{SNR}\;=10\log_{10}(\frac{P_{signal}}{P_{noise}})$$

**Figure 14:**
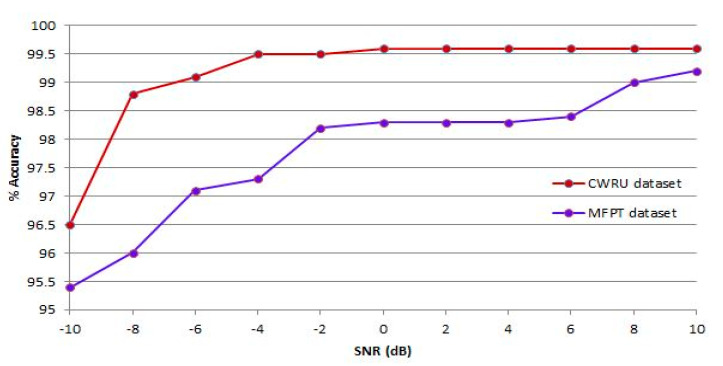
Evaluation in a noisy situation.

The proposed system is validated with noisy signals. Figure 14 depicts the performance of the proposed system in noisy situations with SNR values ranging from −10 dB to 10 dB. The red line in Figure 14 shows classification effectiveness during a noisy environment with SNR values between −10 dB and 10 dB, while the blue line shows classification performance in the same environment with the same SNR values. However, even in a noisy environment with SNR = −10 dB, the proposed system exhibits a high accuracy of 96.50% on the CWRU dataset and 95.40% on the MFPT dataset. The outcomes of this experiment indicate that the proposed system is strong and capable of handling noisy environments.

#### 3.2.4. Performance Evaluation with Various Loads

**Figure 15:**
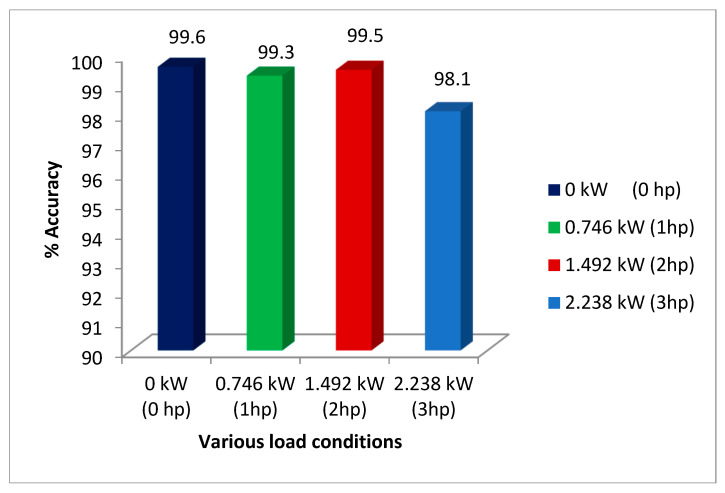
Performance evaluation with various loads.

Machines and their bearings have to operate under a variety of load conditions in the field or in real-world applications. It is more difficult to diagnose faults when the vibration signal’s characteristics alter in response to variations in load. Vibration signals under various loads of 0 kW (0 hp), 0.746 kW (1 hp), 1.492 kW (2 hp), and 2.238 kW (3 hp) are used to evaluate the proposed system after training. The achieved findings are highly efficient, with accuracy ranging from 98.10% to 99.60%. The results are shown in Figure 15. The results obtained indicate that the proposed system performs superiorly under various loading situations.

## 4. Conclusions

Systems for machine fault detection and diagnosis have widely used DL models. However, the fully connected layer of AlexNet has the problem of too many training parameters, which increases the training and testing time and causes overfitting. The effectiveness of intelligent defect diagnosis techniques suffers significantly due to the constantly shifting working load and the inevitable noise from the location of operation. In the proposed technique, the best pre-trained CNN model is selected for the bearing fault diagnosis. The AlexNet model is modified by replacing the FC layer with a GAP layer and adding some BN layers to prevent this internal covariate shifting, which effectively decreases the parameter quantity, overfitting, and calculating the time of the CNN model. Additionally, a hybrid model concept is made to achieve the best performance. The proposed modified AlexNet–SVM hybrid model can achieve an accuracy of 99.60% on the CWRU and can accurately identify bearing faults under various load conditions as well as noisy environments with changing SNR values. The proposed approach is capable of classifying bearing defects under various load conditions as well as in noisy situations.

## Figures and Tables

**Table 1:** Classification of the results of the pre-trained model.

Pre-Trained Model	Accuracy (%)					Average
	Trial 1	Trial 2	Trial 3	Trial 4	Trial 5	
GoogleNet	95.30	94.98	94.65	95.60	95.80	95.27
VGG16	87.50	87.98	88.50	87.30	88.50	87.96
**AlexNet**	**97.50**	**97.30**	**98.10**	**96.88**	**97.78**	**97.51**
ResNet50	96.98	97.35	96.38	95.88	96.78	96.67

**Table 2:** Number of parameters of the AlexNet model.

Layer Name	Size	Parameters
conv1	55 × 55 × 96	34,944
conv2	27 × 27 × 256	614,656
conv3	13 × 13 × 384	885,120
conv4	13 × 13 × 384	1,327,488
conv5	13 × 13 × 256	884,992
fc6	4096 × 1	37,752,832
fc7	4096 × 1	16,781,312
fc8	1000 × 1	4,097,000
Total number of parameters		62,378,344

**Table 3:** Hyper-parameter descriptions.

Name	Type	Activations	Learnable
Data 227 × 227 × 1 images	Image input	227 × 227 × 1	-
conv1	Convolution	55 × 55 × 96	Weights 11 × 11 × 1 × 96; Bias 1 × 1 × 96
batchnorm-1	Batch Normalization	55 × 55 × 96	offset 1 × 1 × 96 scale 1 × 1 × 96
Relu-1	ReLu	55 × 55 × 96	-
Pool-1	Max Pooling	27 × 27 × 96	-
conv2	Convolution	27 × 27 × 256	Weights 5 × 5 × 48 × 128; Bias 1 × 1 × 128 × 2
batchnorm-2	Batch Normalization	27 × 27 × 256	offset 1 × 1 × 256 scale 1 × 1 × 256
Relu-2	ReLu	27 × 27 × 256	-
Pool-2	Max Pooling	13 × 13 × 96	-
conv3	Convolution	13 × 13 × 384	Weights 3 × 3 × 256 × 384; Bias 1 × 1 × 384
batchnorm-3	Batch Normalization	13 × 13 × 384	offset 1 × 1 × 384 scale 1 × 1 × 384
Relu-3	ReLu	13 × 13 × 384	-
conv4	Convolution	13 × 13 × 384	Weights 3 × 3 × 192 × 192; Bias 1 × 1 × 192 × 2
batchnorm-4	Batch Normalization	13 × 13 × 384	offset 1 × 1 × 384 scale 1 × 1 × 384
Relu-4	ReLu	13 × 13 × 384	-
conv5	Convolution	13 × 13 × 256	Weights 3 × 3 × 192 × 128 × 2; Bias 1 × 1 × 128 × 2
batchnorm-5	Batch Normalization	13 × 13 × 256	offset 1 × 1 × 256 scale 1 × 1 × 256
Relu-5	ReLu	13 × 13 × 256	-
Pool-5	Max Pooling	6 × 6 × 256	-
drop 6	Dropout	6 × 6 × 256	-
Relu-6	ReLu	6 × 6 × 256	-
gapool	Global Average Pooling	1 × 1 × 256	-
Prob Softmax	Softmax	1 × 1 × 4	-

**Table 4:** Number of parameters of the modified AlexNet model.

Layer Name	Size	Parameters
conv1	55 × 55 × 96	34,944
Batchnorm-1	55 × 55 × 96	384
conv2	27 × 27 × 256	614,656
Batchnorm-2	27 × 27 × 256	1024
conv3	13 × 13 × 384	885,120
Batchnorm-3	13 × 13 × 384	1536
conv4	13 × 13 × 384	1,327,488
Batchnorm-4	13 × 13 × 384	1536
conv5	13 × 13 × 256	884,992
Batchnorm-5	13 × 13 × 256	1024
Total number of parameters		3,752,704

**Table 5:** Impact on global average pooling (GAP).

AlexNet	Using Fully Connection Layer (FC)		Using Global Average Pooling (GAP)	
	Training Accuracy (%)	Testing Accuracy (%)	Training Accuracy (%)	Testing Accuracy (%)
Trial 1	97.50	94.40	95.80	95.20
Trial 2	97.30	94.30	95.50	95.10
Trial 3	98.10	95.20	96.60	96.00
Trial 4	96.88	94.00	95.20	94.85
Trial 5	97.78	94.50	96.20	95.95

**Table 6:** Comparison of results between the traditional AlexNet and modified AlexNet.

Method	Training Accuracy (%)	Testing Accuracy (%)	Training Time(s)	Testing Time (s)
AlexNet	97.50	94.40	148.92	0.295
Modified AlexNet	98.74	98.30	112.48	0.157

**Table 7:** Evaluation results of the proposed system.

Model	TP	FP	FN	Precision Rate	Recall Rate	F1-Measure
Modified AlexNet-SVM	185	2	0	98.93%	100%	99.46%
Modified AlexNet-Softmax	183	3	1	96.51%	98.81%	98.92%
Modified AlexNet-kNN	186	3	0	98.41%	100%	99.20%

## Data Availability

“CWRU dataset” at https:// engineering.case.edu (accessed on 28 October 2021), and “MFPT dataset” at https:// www.mfpt.org/fault-data-sets (accessed on 28 October 2021).
